# Characterisation of Bovine Leukocyte Ig-like Receptors

**DOI:** 10.1371/journal.pone.0034291

**Published:** 2012-04-02

**Authors:** Louise Hogan, Sabin Bhuju, Des C. Jones, Ken Laing, John Trowsdale, Philip Butcher, Mahavir Singh, Martin Vordermeier, Rachel L. Allen

**Affiliations:** 1 Centre for Infection, Division of Clinical Sciences, St George’s, University of London, Cranmer Terrace, London, United Kingdom; 2 TB Research Group, Animal Health and Veterinary Laboratories Agency (AHVLA), Weybridge, New Haw, United Kingdom; 3 Department of Gene Regulation and Differentiation, Helmholtz-Zentrum für Infektionsforschung, Braunschweig, Germany; 4 Immunology Division, Department of Pathology, University of Cambridge, Cambridge, United Kingdom; 5 LIONEX Diagnostics & Therapeutics, Braunschweig, Germany; California State University Fullerton, United States of America

## Abstract

Leukocyte Immunoglobulin-like receptors (LILR) are innate immune receptors involved in regulating both innate and adaptive immune functions. LILR show more interspecies conservation than the closely related Killer Ig-like receptors, and homologues have been identified in rodents, primates, seals and chickens. The murine equivalents, paired Ig-like receptors (PIR), contain two additional immunoglobulin domains, but show strong sequence and functional similarities to human LILR. The bovine genome was recently sequenced, with preliminary annotations indicating that LILR were present in this species. We therefore sought to identify and characterize novel LILR within the *Bos taurus* genome, compare these phylogenetically with LILR from other species and determine whether they were expressed *in vivo*. Twenty six potential bovine LILR were initially identified using BLAST and BLAT software. Phylogenetic analysis constructed using the neighbour-joining method, incorporating pairwise deletion and confidence limits estimated from 1000 replicates using bootstrapping, indicated that 16 of these represent novel bovine LILR. Protein structures defined using protein BLAST predict that the bovine LILR family comprises seven putative inhibitory, four activating and five soluble receptors. Preliminary expression analysis was performed by mapping the predicted sequences with raw data from total transcript sequence generated using Genome Analyzer IIx (Illumina) to provide evidence that all 16 of these receptors are expressed *in vivo*. The bovine receptor family appears to contain receptors which resemble the six domain rodent PIR as well as the four domain LILR found in other species.

## Introduction

Members of the Leukocyte Immunoglobulin-like receptor (LILR) family are potent modulators of immune function. Through their expression on monocytic cells, LILR can regulate Toll Like Receptor (TLR) activity [1], [2] and antigen presenting phenotype [3], [4], [5], [6]. This ability to regulate both innate and adaptive immune functions of antigen presenting cells indicates that LILR play a pivotal role in shaping immune responses. Accordingly, LILR activity has been shown to influence the immune response during bacterial and viral infection, determining the outcome of disease [2], [7], [8].

The human LILR family consists of eleven functional genes and two pseudogenes [9], [10], [11] encoded within the leukocyte receptor complex (LRC) on chromosome 19q13.4 [10], [12]. LILR belong to the Ig superfamily, and each possess between 2–4 extracellular C2-type Ig domains [13] and a type I transmembrane domain [14]. The IgC2 domains within the human LILR family are referred to as D1-D4.

The 11 receptors of the human LILR family have been subgrouped on the basis of their signalling ability and their ligand specificity. Receptors classified as inhibitory (LILRB1-5) have a cytoplasmic tail containing 2–4 immunoreceptor tyrosine-based inhibitory domains (ITIMs). Activating LILR (LILRA1, 2, 4–6) lack any signalling motif of their own, but instead possess a charged arginine residue which enables association with ITAM encompassing adaptor proteins such as FcεRIγ [15]. LILRA3, whose coding sequence contains no transmembrane or signalling domains, is expressed as a soluble molecule and is absent from some haplotypes [11], [16]. Despite their designation as activating, receptors which signal through ITAM domains are capable of exerting inhibitory effects on cell function [15], [17], [18].

LILR orthologues are found in rodents where they are known as the paired immunoglobulin-like receptors (PIR). The PIR family contains multiple activating receptors (PIR-A) but only one inhibitory receptor, PIR-B [19], [20], [21]. A further murine homologue, LILRB4 (previously known as gp41B1), also exists [19], [20], [21], [22], [23], [24]. PIR contain six Ig domains [19], but share sequence similarity with the D1-D4 domains in LILR, as well as expression profile, functional effects and ligand specificity for MHC-I, to the extent that the murine receptors are also capable of recognising human MHC-I [25], [26].

The preservation of two structurally similar receptor groups, LILR and PIR, indicates that these receptors and their immune functions may be highly conserved through evolution. This is in contrast to the closely-related KIR, for which a structurally unrelated family of receptors known as Ly49 has evolved in some species to perform the same functional role [27]. To date, LILR homologues and LILR like sequences have been identified in humans [28], [29], [30], rodents [22], chimpanzees [13], gray seals [27] and chickens [31], [32], [33]. We sought to investigate the presence of LILR genes and their expression in the domesticated cow (*Bos Taurus*). Due to the high level of LILR conservation between species, we hypothesised that the structure and function of bovine LILR will resemble that of human LILR. In support of this, bovine KIR homologues have been identified, and their structure and function, but not location, were found to be homologous to that of human KIR [34], [35], [36].

The bovine genome has recently been sequenced and published [34], and 35 predicted bovine LILR have been annotated (Table 1). Of these 35 predicted bovine LILR, 17 were annotated on NCBI GenBank using the gene prediction method GNOMON, and the remaining 18 receptors were annotated on Ensembl, using Genebuild. Table 1 details the gene ID, transcript ID, sequence length of each previously predicted bovine LILR, and which human receptor they are thought to be orthologous to. These sequences are mapped to the same region of bovine chromosome 18 (chr18:62833056-63654437), with the exception of two sequences mapped to position chr18:1073938-1097100 and seven Ensembl sequences which have been mapped to three currently unknown contigs (Un.004). As many of these sequences show considerable overlap, and information is limited regarding their protein structure or relationship to human LILR, we sought to further analyse these sequences and search the bovine genome for other potential LILR. Here, we address whether these predicted bovine LILR sequences have been adequately annotated and further describe the identification, classification and expression of 16 novel bovine LILR transcripts in bovine immune cell subsets.

**Table 1:** This table depicts the gene ID, transcript ID, sequence length and location, and which human receptor each predicted bovine LILR is thought to be orthologous to.

Source	Gene ID	Transcript ID	Bp	Human LILR
Ensembl:Genebuild	ENSBTAG00000000930	ENSBTAT00000001231	675	Pseudo
	ENSBTAG00000037830	ENSBTAT00000056991	579	Novel
	ENSBTAG00000038797	ENSBTAT00000054607	840	Novel
	ENSBTAG00000038797	ENSBTAT00000057210	636	Novel
	ENSBTAG00000038797	ENSBTAT00000052297	792	Novel
	ENSBTAG00000019547	ENSBTAT00000026050	900	Novel
	ENSBTAG00000019547	ENSBTAT00000050632	675	Novel
	ENSBTAG00000038828	ENSBTAT00000010878	1947	Novel
	ENSBTAG00000038828	ENSBTAT00000025268	1899	Novel
	ENSBTAG00000038828	ENSBTAT00000051925	1278	Novel
	ENSBTAG00000008266	ENSBTAT00000001308	2484	Novel
	ENSBTAG00000005841	ENSBTAT00000026048	1794	Novel
	ENSBTAG00000026944	ENSBTAT00000025766	2034	Novel
	ENSBTAG00000019348	ENSBTAT00000007679	2484	Novel
	ENSBTAG00000009801	ENSBTAT00000018868	1923	Novel
	ENSBTAG00000009801	ENSBTAT00000052581	1254	Novel
	ENSBTAG00000009801	ENSBTAT00000012925	1260	Novel
	ENSBTAG00000039413	ENSBTAT00000028085	1839	Novel
NCBI GenBank:GNOMON	LOC790181	XM_001788807.2	1548	LILRB2
	LOC100335479	XM_002684435.1	1932	LILRB2
	LOC790074	XM_002684434.1	1506	LILRB5
	LOC100139209	XM_001787176.2	1653	LILRB5
	LOC100139766	XM_001788788.2	2100	LILRA6
	LOC618416	XM_002684433.1	2232	LILRB5
	-	XM_002684429.1	1205	LILRB5/ LILRA4
	-	XM_870741.4	1476	LILRB5/ LILRA4
	LOC790077	XM_001256651.3	2006	LILRB5
	-	XM_001256691.3	2358	LILRB3
	-	NM_001102357.1	1152	LILRA4
	LOC510520	XM_002684432.1	1746	LILRA6
	LOC507024	XM_583568.5	3639	LILRB5
	LOC786365	XM_001250913.3	8704	PIRA4
	LOC100336589	XM_002695415.1		LILRA2
	LOC100301222	XR_083953.1		LILRA2

## Methods

### Bioinformatic Identification of Bovine LILR Homologues

Coding sequences (CDS) and the corresponding peptide sequences were obtained from the Consensus CDS (CCDS) database available through Ensembl (http://www.ncbi.nlm.nih.gov/CCDS/) for all human LILR and KIR, bovine KIR, murine PIR and key primate LILR. These sequences were then used to search all current bovine genome assemblies (Project reference AAFC03000000) through the *Bos taurus* nucleotide BLAST programme available through NCBI (http://www.ncbi.nlm.nih.gov/genome/seq/BlastGen/BlastGen.cgi?taxid=9913). As LILR contain Ig domains, which are found in many other proteins, the data obtained from BLAST were confirmed, and the chromosomal locations were clarified using the BLAST-Like Alignment Tool (BLAT) (UCSC Genome Bioinformatics, USA, http://genome.ucsc.edu/cgi-bin/hgBlat, [37]). The genomic DNA identified as encoding potential bovine LILR was then analysed using GeneScan (http://genes.mit.edu/GENSCAN.html, [38]) or GeneMark (http://exon.gatech.edu/eukhmm.cgi [39]), to predict the CDS and resulting peptides, and the predicted protein domains and exon boundaries were obtained using protein BLAST, available through NCBI (http://blast.ncbi.nlm.nih.gov/Blast.cgi?PAGE=Proteins), and Ensembl respectively.

### Sequence Analysis

Sequence comparisons of the predicted CDS were analysed using Clustalw, a multiple sequence alignment tool, in which the results are given as a percentage of identity to pairwise alignments [40].

### Phylogenetic Analysis

Phylogenetic analysis of the amino acid sequences for the individual Ig domains was performed using MEGA5 software [41], and constructed using the neighbour-joining method [42], incorporating pairwise deletion and confidence limits estimated from 1000 replicates using bootstrapping [43]. Pairwise deletion was used as there is a high number of sequences to be analysed and complete deletion would have resulted in a significant reduction in the amount of each sequence available for analysis.

### Prediction of Transmembrane Helices

Transmembrane regions were mapped using the TMHMM Server v. 2.0 available at the Center for Biological Sequence Analysis, Technical University of Denmark, and the arginine residues and ITIMs were mapped to each peptide sequence, using the established V/L/S/NxYxxL/V ITIM motif [44].

### Expression Analysis

To determine whether mRNA transcripts corresponding to our newly identified sequences are expressed, cDNA transcripts generated from RNA isolated from cultured peripheral blood cells of cows were mapped to the putative LILR sequences. Purification of mRNA from total RNA, cleavage of mRNA, cDNA synthesis and library preparation were performed according to manufacturer's standard protocols using the mRNA-Seq-8 Sample Preparation Kit (Illumina). Cluster generation, primer hybridization and sequencing reactions were performed following the manufacturer’s recommended protocols [45]. Reads with an expected read length of 36-bases were generated from each library using a Genome Analyzer IIx (Illumina).

To evaluate the best prediction server for each corresponding bovine LILR, the chromosomal sequences chr18:62832447-63370336, Un.004.625:2160-77036, and Un.004.1339:17781-25700 extracted from the bovine genome were used as reference for mapping. Mapping was performed using CLC Genomics Workbench 4.7.2 with mismatch cost of 2 using ungapped alignment. Positional mapping of each base for all mappable reads was calculated using custom designed Perl script using the SAM file. Each chromosomal segment containing predicted sequences was inspected with Artemis [46] using the predicted annotations obtained based on each prediction server and the positional mapping of reads. The predicted exonic sequence was further inspected and supported by the presence of regions with a high density of mappable reads from the RNA-sequencing. The most likely prediction was selected based on the combined annotation and RNA-sequence analysis for each bovine LILR. The RNA-sequence reads were re-mapped using the same reference sequence but with a lower mismatch cost of 1. Regions mapped with more than 99% coverage were considered to represent exonic sequence expressed in peripheral blood cells of cows.

## Results

### Identification of Potential Bovine LILR Transcripts

Using human LILR sequences to search the bovine genome with both BLAST and BLAT, we identified a total of 77 open reading frames (ORF’s) on three contigs as containing potential bovine LILR. Each of these ORF’s was then analysed by GeneScan and GeneMark, to obtain the predicted CDS and corresponding protein sequence, which were then aligned to domains using protein BLAST to identify Ig domains. Of the 77 ORF sequences, 26 were found to contain Ig domains and were selected for further analysis; 19 were located on chromosome 18 between 62,832,497–63,653,564, five sequences were located on unknown contig 004.625 between 2210–76986, and finally, one sequence was found to be located on a further unknown contig 004.1339 between 17831–25650 (data not shown). From this point on, the newly identified transcripts will be referred to as bovine LILR (BL) 1–26.

### Identification of Different Receptor Families

**Figure 1:**
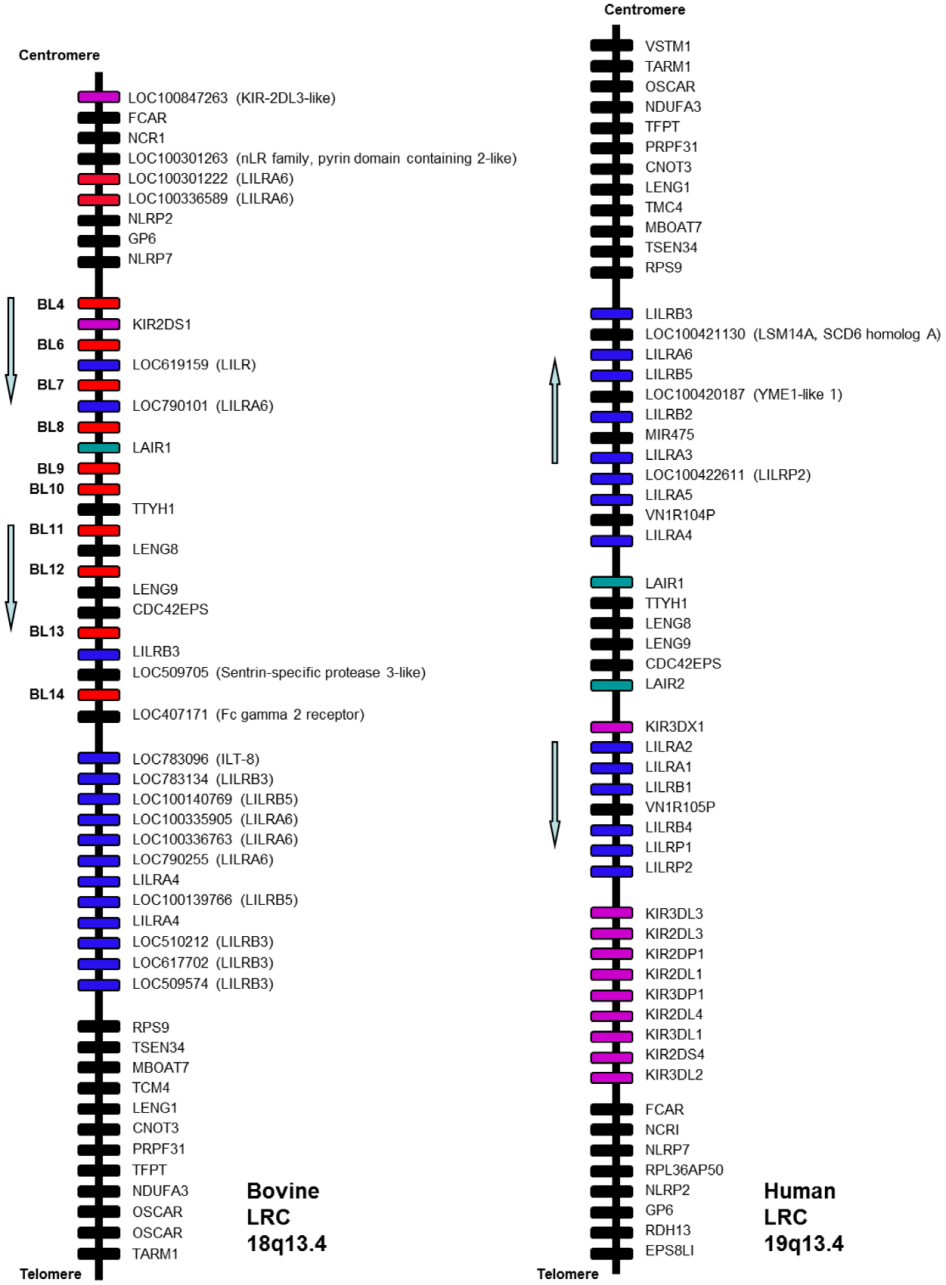
Gene arrangement within the human and bovine LRC. This diagram presents data from NCBI on the locations of predicted and confirmed genes found within the bovine LRC, located on 18q13.4, and compared to the annotated human LRC located on 19q13.4. This data is correct at time of print, although is subject to change due to unallocated genomic scaffolds within the bovine genome. The basic arrangement of the bovine LRC is almost the reverse of the human LRC, with a few exceptions. The KIR and FCaR can be found at the centromeric end of the bovine LRC, whereas in the human LRC they are located at the telomeric end. The LILR are upstream of the KIR in the human LRC, compared to the bovine LRC, where the BL are found downstream of predicted KIR. Furthermore, OSCAR, LENG1, CNOT3 and MBOAT7 are located at the telomeric end of the bovine LRC, compared to the centromeric end of the human LRC.

We then sought to confirm that the sequences identified encode BL rather than other closely related Ig receptors. To do this, we compared the BL to LILR/PIR receptors from other species, including human, murine, bovine, chimpanzee, orang-utan, gorilla and rhesus macaque. Furthermore, as bovine equivalents of other Ig receptors located within the human LRC have also been mapped to this region of chromosome 18 in *Bos taurus*, we extended our search of the annotated sequences to include all LRC sequences mapped within the region of chr18:62832447-63700000. The location of bovine Ig receptors currently mapped to this region, such as the bovine KIR and OSCAR, indicate that the organisation of the bovine LRC is considerably different to that of the human LRC (figure 1) [47]. As proteins encoded within the LRC are structurally and functionally related, it becomes more difficult to isolate and distinguish between one particular set of sequences and another. We therefore also included human and, where available, bovine SIGLEC, OSCAR, KIR, FcGRT, FcaR, LAIR, GpVI and NKp46 in our analysis.

**Figure 2:**
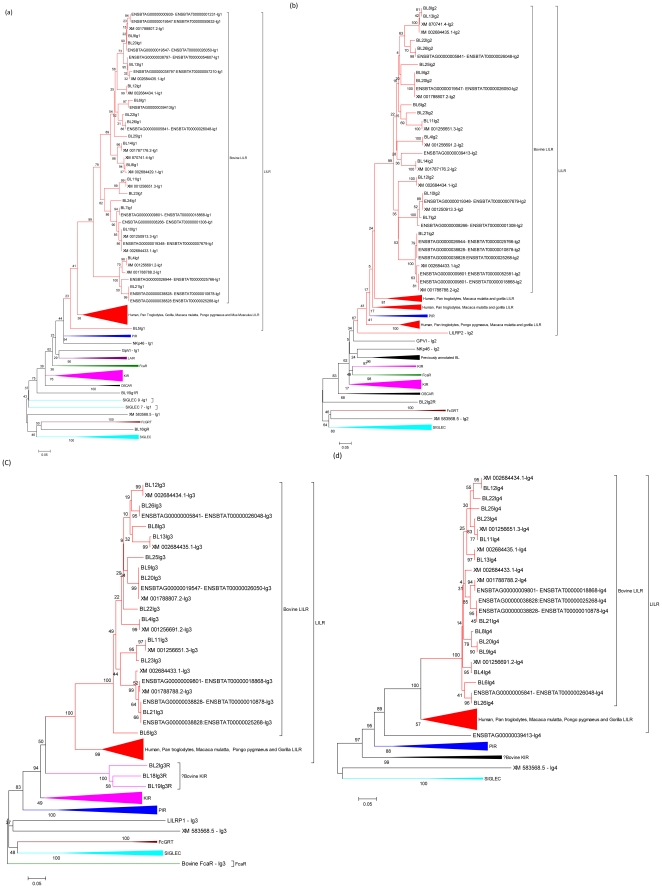
Phylogenetic analysis of Ig domains from receptors located within the LRC of different species. Phylogenetic analysis of Ig domains from receptors located within the LRC of different species: The available Ig domains from SIGLEC, OSCAR, KIR, FcGRT, FcaR, LAIR, GpVI and NKp46 in species including human, murine, bovine, chimpanzee, orang-utan, gorilla and rhesus macaque were analyzed using phylogeny. Phylogenetic trees were constructed using the neighbour-joining bootstrap consensus method inferred from 1000 replicates, pairwise deletion and amino acid p distance. The graphs show Ig1–4 domains (a-d respectively). The receptor groups are labelled with LILR shown in red and PIR in blue.

The exonic regions encoding each Ig domain for all sequences were analysed separately using phylogenetic analysis, which was performed using neighbour-joining bootstrap consensus trees inferred from 1000 replicates, pairwise deletion and amino acid p distance. Because the IgC2 domains fold in a characteristic way for each receptor, and contain a distinct binding site, it is possible to group receptor domains on a functional basis and determine which Ig superfamily receptor group they belong to. The phylogenetic analyses of these domains are shown in figures 2a-d.

From the 26 putative LILR transcripts, 16 receptors are closely associated with LILR transcripts from other species. The bovine LILR appear to have evolved in a distinct manner compared to that of other species, but bovine LILR do not appear to be as divergent as murine PIR are to primate LILR. There are six transcripts (BL1, BL2, BL17, BL18, BL19 and BL24) which appear to be novel genes that closely resemble KIR transcripts, although whether these genes are novel KIR sequences is an exact distinction that can not be made from this data. BL3 resembles the already annotated bovine LAIR-1 gene, however it is mapped to a different region on chromosome 18. This could be due to recent rearrangements in the bovine genome where an effort has been made to position unallocated genomic scaffoldings. Two transcripts, BL15 and BL16, did not cluster with any groups of receptors in this analysis. And finally, BL5 Ig1 is more closely associated with LILR than the other Ig receptor groups analysed, however, it appears to be an outlier within the LILR group, and the Ig2 domain is not closely related to any other group of receptors. Therefore, further investigation is required to determine exactly what type of receptor this transcript encodes.

Interestingly, in our analysis (data not shown) the previously annotated bovine LILR XM_002684432.1, ENSBTAT00000012925, ENSBTAT00000051925, ENSBTAT00000056991 and the novel Gorilla LILR ENSGGOT00000031548 are all more closely grouped with the KIR family than LILR, and contain 1–3 Ig domains, which is more characteristic of the KIR genes. Moreover, although the Ig2 domain of ENSBTAT00000052581 is clustered within the LILR transcripts, the Ig1 and Ig3 domains again are more closely associated with the KIR group. BL5 together with ENSBTAT0000001231, ENSBTAT00000050632, ENSBTAT00000054607, ENSBTAT00000057210, ENSBTAT00000052297 and XM_2684429 are clustered separately from the other receptor groups, despite all these transcripts (with the exception of ENSBTAT00000052297) having an Ig1 domain that matches the LILR group. These receptors only contain two Ig domains, and may represent a separate receptor family, or a group of very divergent LILR.

### Comparison of Novel Sequences with Previously Predicted Sequences

**Figure 3:**
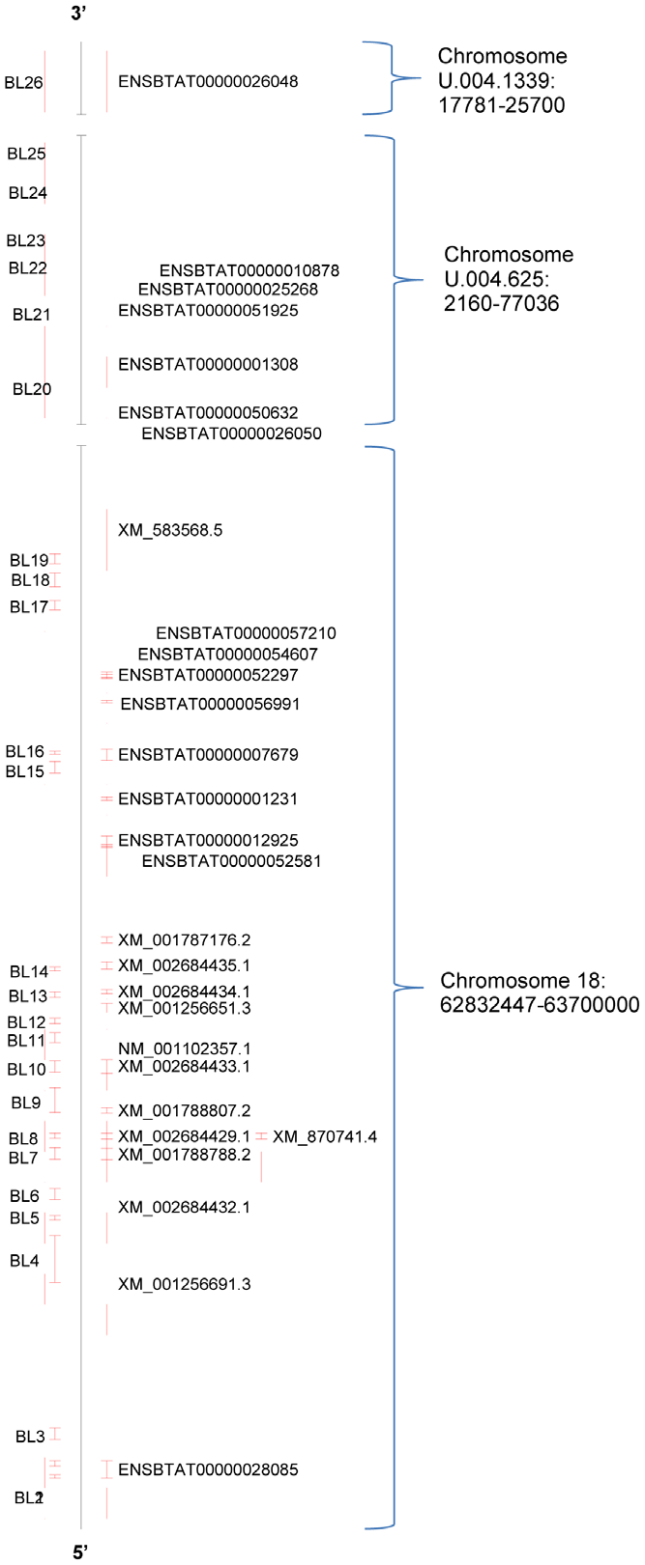
Chromosomal locations for predicted bovine LILR. Chromosomal locations for bovine LILR: Bovine LILR have been mapped to compare the sequence locations of those found in our search compared to the previously annotated sequences.

We then sought to ascertain whether any of the sequences identified in our search were identical to any of the previously annotated sequences. Several comparisons were made to determine whether any duplications were present;, the chromosome position for each sequence was mapped, whole sequences were compared using clustalw, and individual Ig domains were phylogenetically analysed as described above. Three of the previously annotated sequences, XM_002695415.1, NM_001102357.1 and XR_083953.1, were excluded from the phylogenetic analysis, as these sequences could not be mapped within the specified chromosomal regions, and therefore their exonic regions could not be fully determined. A chromosome map is shown in figure 3, and the phylogenetic analysis is shown in Figure S1a-f and summarized in table 2.

**Table 2:** Summary of phylogenetic analysis in figures 2a-f, indicating where transcripts contained identical Ig domains.

	New LILR Sequences					
Transcript ID	BL4	BL10	BL11	BL12	BL21	BL26
5841–26048						*
19348–7679		*				
38828–25268					*	
38828–10878					*	
XM_002684434.1				*		
XM_001250913.3		*				
XM_001256651.3			*			
XM_001256691.2	*					

The chromosome map shown in figure 3 demonstrates that several of the previously annotated bovine LILR overlap at least partially with those found in our search. Although ENSBTAT00000026048, XM_002684433.1, XM_001788788.2 and XM_002684429.1 overlap with BL26, BL10, BL7, BL8 and XM_870741.4 respectively, clustalw analysis showed that none of these overlapping sequences had a similarity of ≥95% (data not shown). However, it is interesting to note that XM_002684429.1 and XM_870741.4 are mapped to the same position and have an alignment score of 95%, and are therefore possibly two alternatively spliced transcripts of the same gene.

In our combined analysis, the clustalw results (data not shown), indicated that a total of 85 sequences were ≥95% similar to one or more other sequences. Of these, 41 of the previously annotated sequences were ≥95% similar to other annotated sequences, 32 previously annotated sequences were ≥95% similar to a newly identified BL sequence, and 12 of the newly identified BL sequences were ≥95% similar to another newly identified BL sequence. These results were expected as LILR are very similar in structure and function both in humans and between species, and some receptors may differ only in their ligand binding sites. BL14 was found to be identical to both XM_001787176.2 and ENSBTAT00000052297 along the entire sequence length, however, it is worth noting that BL14 and ENSBTAT00000052297 have short sequence lengths of 945aa, and 840aa respectively, which might account for the increased identity matches. Furthermore, these sequences are located in three different positions on the chromosome.

Each BL transcript contains up to 6 Ig domains. Phylogenetic analysis (figure S1 a-f and table 2) demonstrated that between our 16 BL transcripts there were a total of 17 Ig domains present that were also identified amongst the previously annotated transcripts. There were six of the newly identified sequences for which all of the Ig domains could be matched to all the Ig domains encoded in another annotated transcript, and in two cases these new BL sequences were matched entirely to two different annotated sequences. From the six new sequences, three of the matches, BL10, BL11 and BL12, are located in different non-overlapping positions on the chromosome. BL4 partially overlaps XM_007256691.2, and therefore the Ig domains for these two receptors may be identical, although in our search we identified a different ORF. Finally, BL21 was shown to share identical Ig domains with both ENSBTAT00000025268 and ENSBTAT00000010878, which are two transcripts from the same gene, and BL26 has identical Ig domains to ENSBTAT00000026048. These transcripts are located in the same chromosomal positions, but, as the clustalw results indicated BL21 had a 75% and 74% identity to ENSBTAT00000025268 and ENSBTAT00000010878 respectively, and BL26 had an 85% identity to ENSBTAT00000026048. Consequently, we concluded that our newly identified transcripts are significantly different from those previously reported. However, it cannot be ruled out that they are different transcripts of the same gene. Therefore, from the results presented here, we suggest that all transcripts identified in our search are novel, and have not been previously reported.

### Structure of Novel BL

**Figure 4:**
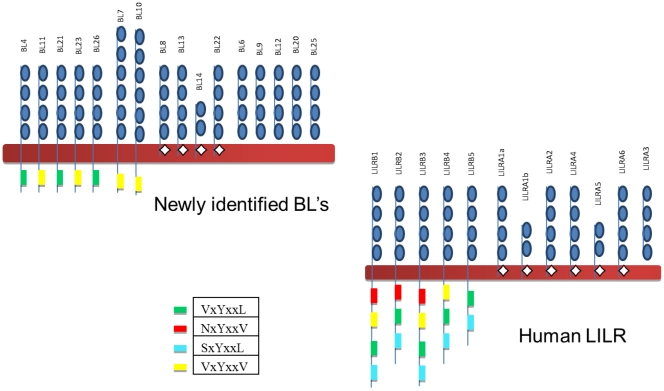
Predicted structures of bovine LILR compared to that of the known human LILR. Predicted structures of bovine LILR compared to that of the known human LILR: To determine the potential structures of bovine LILR the Ig domains were identified using protein BLAST (NCBI), transmembrane regions were mapped using the TMHMM Server v. 2.0 (Centre for Biological Sequence Analysis, Technical University of Denmark), and the arginine residues and ITIMs were identified and located on each peptide sequence, using the established V/L/S/NxYxxL/V ITIM formula [58].

The predicted structure for the bovine LILR are shown in Figure 4. Based on the presence or absence of ITIMs we predict that there are four activating BL and seven inhibitory BL, a similar repertoire to the six activating and five inhibitory receptors found in humans. We have also identified five receptors within the bovine genome which, on the basis of this analysis, would be predicted to be expressed only in a soluble form. In the human system, LILRA3 is the only genomically encoded soluble receptor, although a common splice site allows most human LILR to be expressed in a soluble form [48]. The signaling domains found in the bovine LILR are more conserved than those found in human LILR. The human receptors contain between 2–4 ITIMs, with VxYxxL and SxYxxL being present on every receptor, whereas the bovine receptors only contain one ITIM on each receptor, either VxYxxV or VxYxxL. It is not clear whether this difference in signaling domains is due to a loss in the bovine genome, or if an extra signaling domain has evolved in primates. It is also possible that human LILR could possess a higher capacity for cellular regulation than bovine LILR, as the Y^-2^ domains have been shown to determine the binding of the cytosolic tyrosine kinases SHP-1 and SHP-2 [49], [50], [51].

### Potential Ligand Binding

Following the solution of a crystal structure for LILRB1 with an HLA-A2 ligand, it was predicted that a subset of LILR (known as group 1), will bind to MHC-I, on the basis of sequence similarities [52]. To establish whether any of our newly identified bovine receptors share ligand specificity, individual exons encoding Ig domains in each sequence were phylogenetically analyzed.

**Figure 5:**
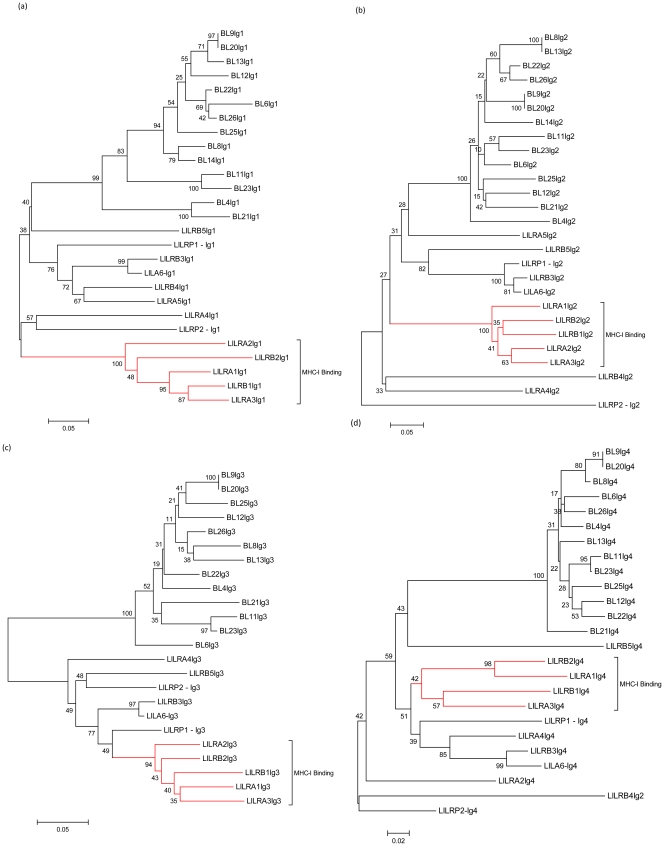
Phylogenetic analysis of bovine and human Ig domains. Phylogenetic analysis of bovine and human Ig domains: The Ig domains of predicted bovine and human LILR were analyzed by constructing neighbour-joining bootstrap consensus trees inferred from 1000 replicates, pairwise deletion and amino acid p distance. The graphs show the comparison of Ig1–4 domains (a-d respectively).

The human group 1 LILR, which share MHC-I ligand specificity, show similar clustering patterns for each of the Ig domains (figure 5a-d) that contain their ligand binding site [53]. No similar clustering patterns were observed for bovine LILR. This indicates that bovine LILR may recognise a broader range of ligands than human LILR.

### PIR-like BL

**Figure 6:**
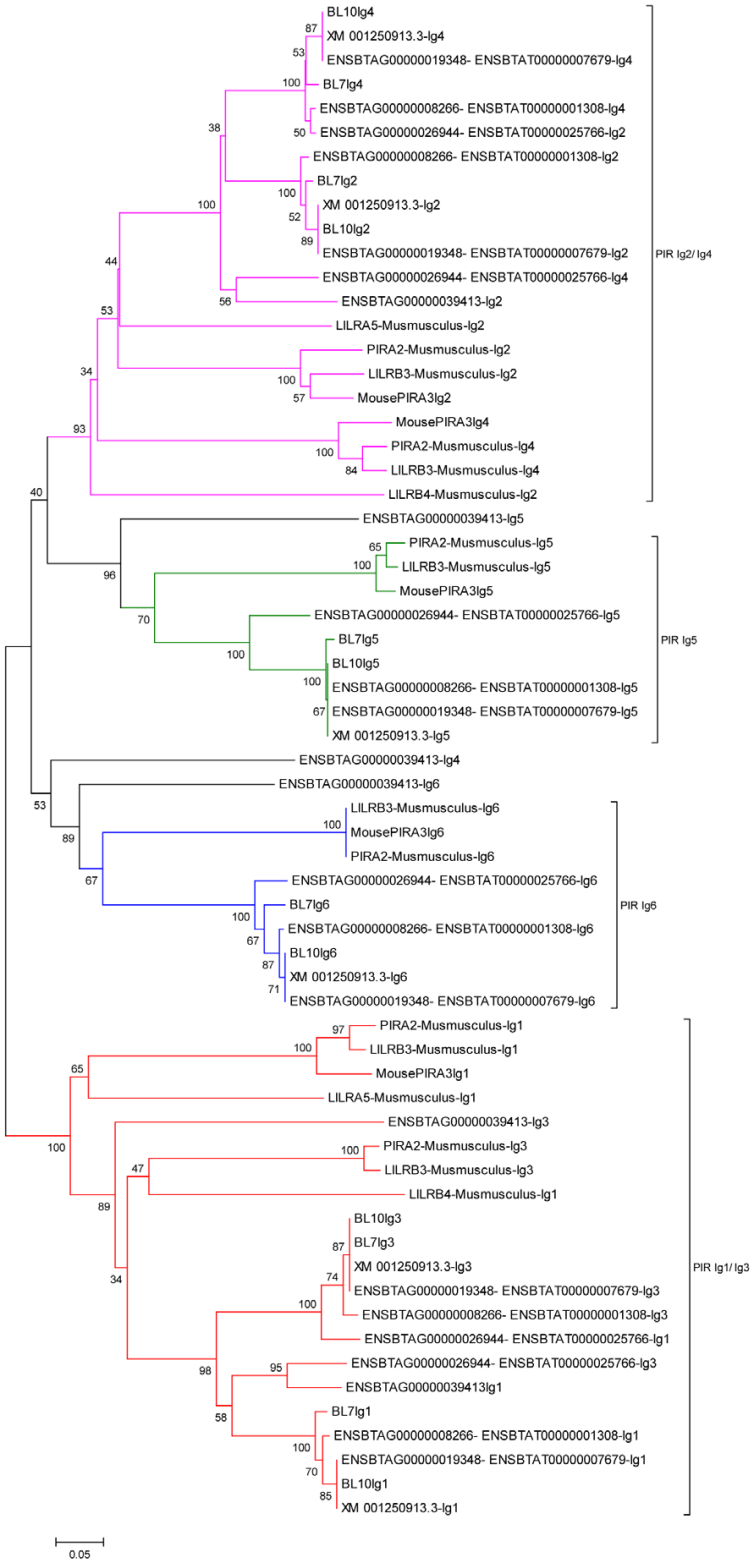
Phylogenetic analysis of murine and predicted bovine PIR Ig domains. Phylogenetic analysis of murine and predicted bovine PIR Ig domains: The Ig domains from murine PIR and the predicted bovine PIR (BL7 and BL10) were analyzed using phylogeny. Phylogenetic trees were constructed using the neighbour-joining bootstrap consensus method inferred from 1000 replicates, pairwise deletion and amino acid p distance. The graph shows the clustering of the individual Ig domains.

Two of the 16 BL transcripts, BL7 and BL10, were more closely clustered with the PIR transcripts, than that of the human LILR. These two transcripts both contain six Ig domains, similar to PIR. Therefore, we repeated our phylogenetic analysis to include all six Ig domains in the PIR-like receptors. The results, shown in figure 6, suggest that *Bos taurus* may possess both LILR and PIR transcripts. This raises the possibility that there is a common ancestral gene for PIR and LILR which has divided to produce two separate lineages, with cattle appearing to have expanded and maintained both. In support of this, Martin, *et al*, have shown that LILR and PIR possess a type I IgC2 domain which is thought to be the most ancient IgC2 domain, from which other domains have in fact evolved [32], [54]. The hypothesis that both PIR and LILR arise from a common ancestral gene is strengthened by the observation that the gorilla LILRA5 homologue, appears to share domains from both PIR and LILR; Ig1 and Ig2 closely resemble that of LILR domains, whereas, the Ig3 and Ig4 closely resemble that of PIR domains (Figures 2a-d and 6). Therefore, it is possible that Bos *taurus* have maintained both receptor groups.

### Expression of BL

Finally, we sought to determine whether any of our newly identified bovine receptors were expressed in bovine PBMC’s. To confirm expression of these genes, we evaluated if they were transcribed in bovine PBMC stimulated with bovine tuberculin PPD. The transcriptome of bovine PBMC was determined by applying Next Generation Sequencing techniques using a Genome Analyzer IIx (Illumina). The resulting sequence data was compared to our predicted BL sequences. As depicted in table 3, we found evidence that all our predicted BL are expressed in PBMC’s, and showed that for six of the 16 predicted BL (BL7, BL10, BL11, BL13, BL22, and BL26) more than 99% of the predicted exonic sequence was mapped, thus validating our in silico predictions.

**Table 3:** Percentage of the predicted BL sequence mapped to the sequence derived by RNA-sequence analysis of Bos taurus peripheral blood cells (PBMCs).

Sequence	% Mapped
BL4	90
BL6	72
BL7	100
BL8	98
BL9	88
BL10	100
BL11	100
BL12	99
BL13	99
BL14	97
BL20	91
BL21	99
BL22	100
BL23	99
BL25	100
BL26	100

## Discussion

In this study, we sought to identify and characterise novel BL using a bioinformatic approach. Our initial search yielded 26 transcripts as potential BL, which were then analysed using phylogeny alongside other structurally similar receptor families also belonging to the Ig-superfamily. By including all available receptor families located on the LRC from as many species as possible, we determined that 16 of these 26 transcripts were LILR like sequences.

The bovine genome is now fully sequenced, and as a result several predicted BL have been previously annotated on NCBI and Ensembl. To ensure our sequences were not duplications of any of these previously reported sequences, we analysed and compared; exonic domains using phylogeny, whole sequences using clustalw, and the mapped position of each sequence. The exonic domains of six of our newly identified sequences were shown to have a high similarity with previously annotated sequences, however the clustalw results and chromosomal location were significantly different. Therefore, we concluded that the 16 BL sequences described here are novel sequences, and have not been previously characterised.

Our results indicate that the bovine genome encodes a total of four activating BL, seven inhibitory BL and five soluble BL, with the possibility of further common splice sites which allow the other BL to be expressed in soluble form. This is a different repertoire of receptors compared to those found in humans, as there is only one human genomically encoded soluble receptor, LILRA3. Soluble receptors, through their nature, are thought to have a higher capacity for cellular regulation, and therefore the presence of five soluble BL in cattle may indicate these receptors have an extended regulatory role. It is worth noting that in humans deficiency of the soluble LILRA3 is associated with Sjogren’s syndrome and multiple sclerosis, indicating an important regulatory role for soluble receptors [55], [56], [57].

As cattle possess more BL it was interesting to find that the inhibitory BL possess fewer signaling domains than those of their human counterparts. The human receptors contain between 2–4 canonical and permissive ITIMs, with VxYxxL and SxYxxL being present on every receptor, whereas the bovine receptors only contain one canonical ITIM on each receptor, either VxYxxV or VxYxxL. This possibly provides a greater diversity of signaling abilities in human LILR than that of BL.

The analysis of human LILR has demonstrated that group I receptors, which all share ligand specificity for MHC I, will cluster together as the exonic Ig domains are highly similar. We analyzed the BL exonic domains to determine if any similar clustering patterns could be identified, which may be indicative of shared ligand specificity. However, in our anaylsis there were no patterns of clustering detected, which may attest to the fact that the BL have a broader range of ligand specificity.

To date, LILR have been reported as highly conserved receptors within all mammalian species, with the exception of PIR in mice. BL appear to have evolved independently to both the PIR and LILR, whilst remaining more conserved than PIR. However, it is interesting to note that the BL genes seem to have retained sequences that resemble both LILR and PIR, which are thought to stem from a common ancestral gene. This is consistent with a sub-section of bovine KIR, described by Guethlein, et al, which are thought to have evolved from a common ancestral gene, KIR3DX [47]. Approximately 136 million years ago, the KIR3D gene is thought to have given rise to two daughter genes, KIR3DL and KIR3DX. Whereas in primates the KIR3DL gene expanded and gave rise to primate KIR, in cattle, the KIR3DX gene is the ancestor for the bovine KIR genes, and in humans this gene is now a non-functional pseudogene [47].

Finally, by analysing bovine PBMC stimulated with bovine tuberculin PPD, we found evidence for the expression of all 16 newly identified BL, six of which we found >99% expressed. Further investigation is required to determine exactly which subsets of PBMC’s each receptor is expressed on, and to elucidate ligand specificities. However, given the role of LILR in humans, we predict that BL will play an important key role in bovine innate immune regulation.

In summary, we have identified 16 novel BL, which appear to have evolved and maintained both LILR and PIR –like sequences from a common ancestral gene. The BL repertoire includes four activating receptors, six inhibitory receptors and five further soluble receptors, six of which we have confirmed the expression of in the peripheral blood cells of *Bos taurus*. The Genbank accession numbers for each sequence are details in table S1. We suggest an appropriate name for these receptors would be Bovine Ig-like Receptors (BIR) (*Table S1)*.

## Supporting Information

Figure S1 **Phylogenetic analysis of newly and previously annotated bovine LILR Ig domains.** *Phylogenetic analysis of Ig domains from predicted bovine LILR: The Ig domains from both the sequences identified in our search and those previously annotated were analyzed by constructing neighbour-joining bootstrap consensus trees inferred from 1000 replicates, pairwise deletion and amino acid p distance. The graphs show the comparison of Ig1–6 domains (a-f respectively) and identical Ig domains are highlighted in red.*
(TIF)

Table S1 **Suggested nomenclature for novel sequences.**
(DOCX)
