# Correction: Characterisation of Bovine Leukocyte Ig-like Receptors

**DOI:** 10.1371/annotation/cfb0e8b5-3815-46c1-997b-c6267ba4856b

**Published:** 2012-08-08

**Authors:** Louise Hogan, Sabin Bhuju, Des C. Jones, Ken Laing, John Trowsdale, Philip Butcher, Mahavir Singh, Martin Vordermeier, Rachel L. Allen

A revised version of Table S1 with additional information can be viewed here: 

[^] 

