# Supplementary figures and images for: Characterisation of Bovine Leukocyte Ig-like Receptors
Source: PLoS One. 2012 Apr 2;7(4):e34291. doi: 10.1371/journal.pone.0034291 (PMC3317502; doi:10.1371/journal.pone.0034291)

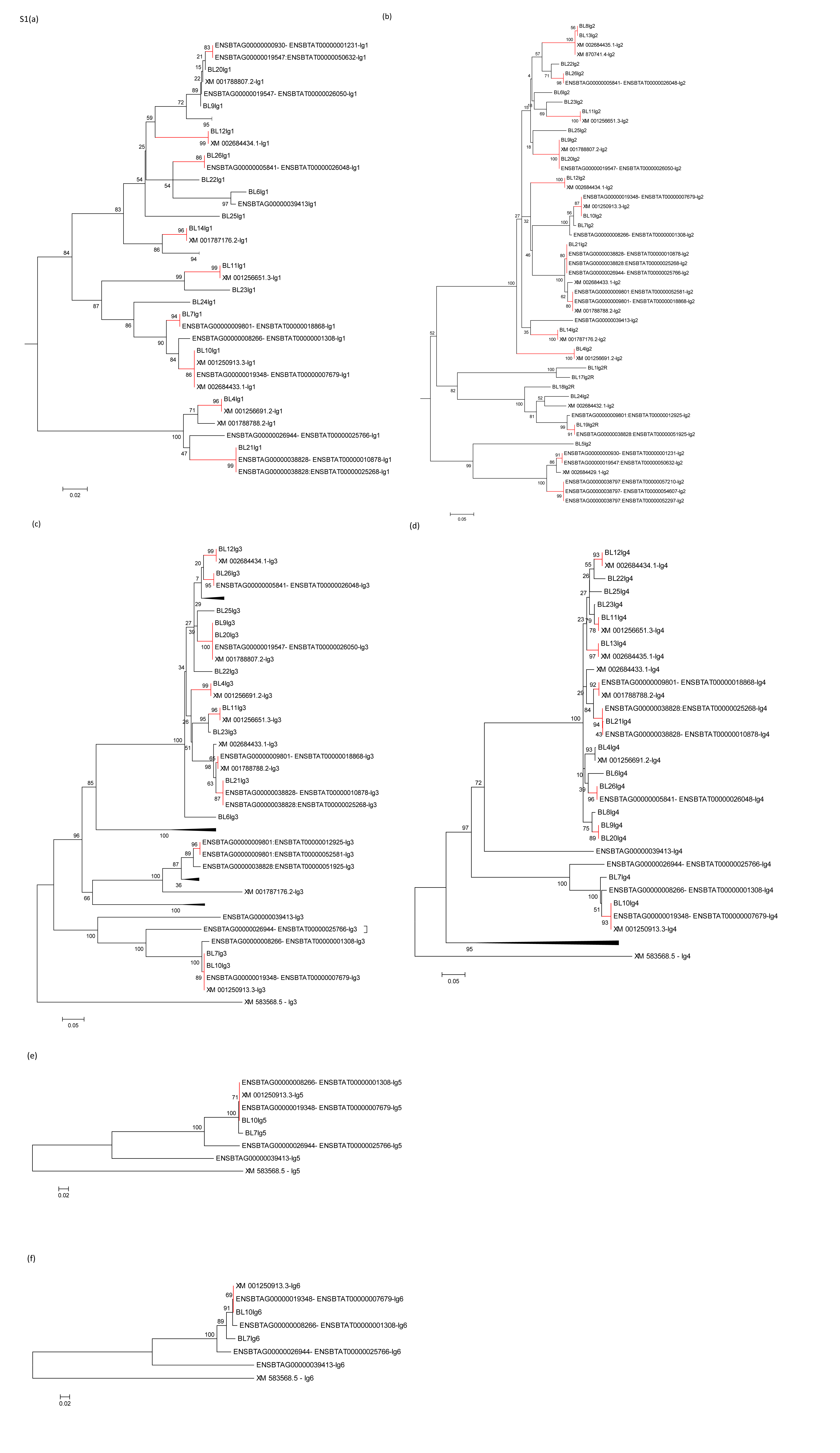

Supplement: Figure S1 — Phylogenetic analysis of newly and previously annotated bovine LILR Ig domains. Phylogenetic analysis of Ig domains from predicted bovine LILR: The Ig domains from both the sequences identified in our search and those previously annotated were analyzed by constructing neighbour-joining bootstrap consensus trees inferred from 1000 replicates, pairwise deletion and amino acid p distance. The graphs show the comparison of Ig1–6 domains (a-f respectively) and identical Ig domains are highlighted in red. (TIF) [file pone.0034291.s001.tif]
