# Supplementary material for: Characterisation of Bovine Leukocyte Ig-like Receptors
Source: PLoS One. 2012 Apr 2;7(4):e34291. doi: 10.1371/journal.pone.0034291 (PMC3317502; doi:10.1371/journal.pone.0034291)
Supplement: Table S1 — Suggested nomenclature for novel sequences. (DOCX) [file pone.0034291.s002.docx]

**S2**

| **Sequence** | **Nomenclature** |
| --- | --- |
| **BL4** | **BIRB1** |
| **BL6** | **BIRS1** |
| **BL7** | **BIRB2** |
| **BL8** | **BIRA1** |
| **BL9** | **BIRS2** |
| **BL10** | **BIRB3** |
| **BL11** | **BIRB4** |
| **BL12** | **BIRS3** |
| **BL13** | **BIRA2** |
| **BL14** | **BIRA3** |
| **BL20** | **BIRS4** |
| **BL21** | **BIRB5** |
| **BL22** | **BIRA4** |
| **BL23** | **BIRB6** |
| **BL25** | **BIRS5** |
| **BL26** | **BIRB7** |
