# Supplementary figures and images for: Correction: Characterisation of Bovine Leukocyte Ig-like Receptors
Source: PLoS One. 2012 Aug 8;7(8):10.1371/annotation/cfb0e8b5-3815-46c1-997b-c6267ba4856b. doi: 10.1371/annotation/cfb0e8b5-3815-46c1-997b-c6267ba4856b (PMC3414627; doi:10.1371/annotation/cfb0e8b5-3815-46c1-997b-c6267ba4856b)

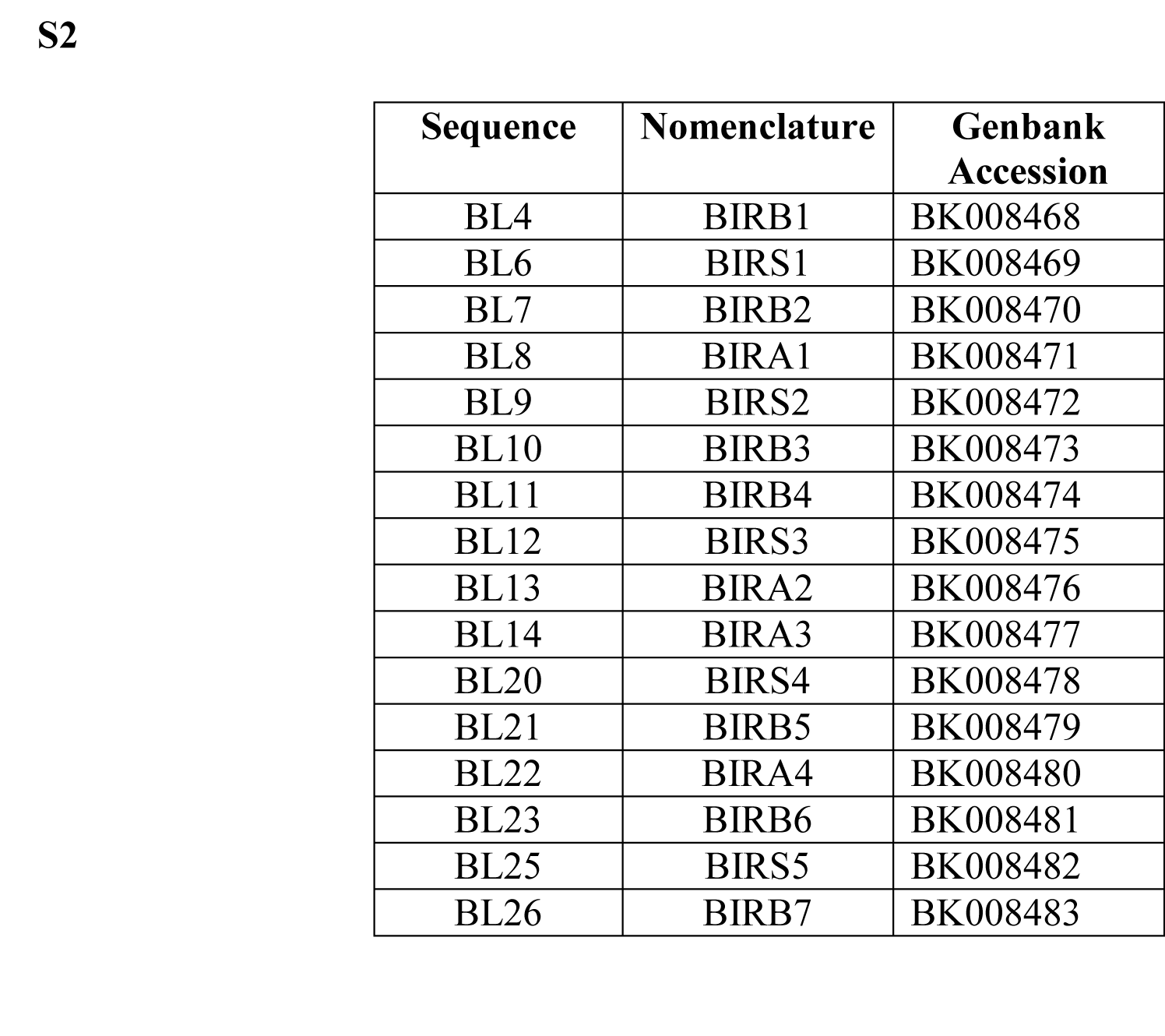

Supplement: Supplementary file 1 [file pone.cfb0e8b5-3815-46c1-997b-c6267ba4856b.s001.tif]
